# CONTROL AT EVERY AGE: HOW EMOTIONAL RESPONSES VARY ACROSS LIFESPAN

**DOI:** 10.1093/geroni/igae098.4146

**Published:** 2024-12-31

**Authors:** Ryan Muskin, David Popivker, Eric Allard

**Affiliations:** Cleveland State University, Cleveland, Ohio, United States; Cleveland State University, Cleveland, Ohio, United States; Cleveland State University, Cleveland, Ohio, United States

## Abstract

Control may play a crucial role in regulating emotional reactivity, with this regulation potentially differing between older and younger adults across various emotional subtypes. Research suggests that perceptions of control tend to decrease with age, which may result in older adults being more sensitive to low-control situations. To explore this, we recruited 60 younger adults (18-35) and 50 older adults (60+) who were asked to relive strong emotional memories involving either loss or failure. Emotional reactivity was assessed through self-reported affect, heart rate variability, and electrodermal activity. Our findings support the hypothesis that as perceived control increases, emotional reactivity decreases for older adults but increases for younger adults (p <.05). Further analysis of discrete emotions revealed a positive correlation between perceived control and positive affect for older adults (Pearson’s r =.34), but not for younger adults. These results suggest that lower perceived control in older adults may lead to greater emotional reactivity and reduced positive affect. Future research should investigate how the interplay between age, control, and emotions influences emotion regulation strategies, particularly those that involve accepting diminished control.

